# Carotid Endarterectomy for a Case with an Extremely Twisted Internal Carotid Artery

**DOI:** 10.31662/jmaj.2023-0033

**Published:** 2023-09-20

**Authors:** Sogo Oki, Masaki Ito, Masayuki Gekka, Tomohiro Yamauchi, Miki Fujimura

**Affiliations:** 1 Department of Neurosurgery, Tomakomai City Hospital, Tomakomai, Japan; 2 Department of Neurosurgery, Hokkaido University Graduate School of Medicine, Sapporo, Japan; 3 Department of Neurosurgery, Otaru General Hospital, Otaru, Japan

**Keywords:** anatomical variation, carotid endarterectomy, carotid stenosis, twisted internal carotid artery, vascular disorder

## Abstract

The internal carotid artery (ICA) typically runs posterolaterally to the external carotid artery (ECA) at the level of the common carotid artery (CCA) bifurcation in the neck. The “twisted ICA” is an anatomical variation, wherein the ICA is medial to the ECA. Several studies on the twisted ICA have discussed its anatomical definition, incidence, clinical features, and surgical results in patients with luminal stenosis. Computed tomography angiography (CTA)-based analyses of surgically treated cohorts documented a twist angle, reaching up to 95°. Carotid endarterectomy (CEA) was successfully performed for these patients. This study reports a case of a significantly twisted ICA with severe luminal stenosis that was successfully treated with CEA. An 81-year-old male was incidentally diagnosed with asymptomatic right ICA stenosis based on magnetic resonance (MR) angiography. Three-dimensional (3D)-CTA showed that the ICA revealed 74% stenosis of the ICA, based on the North American Symptomatic Carotid Endarterectomy Trial criteria. The 3D-CTA showed the ICA medial to the ipsilateral ECA at the level of the CCA bifurcation in the neck. It extended proximally to the pharynx, and the twist angle was 102°. Black-blood MR of the carotid plaque exhibited a high intensity on T1-weighted imaging, indicating vulnerability. Intraoperatively, the position of the ICA was corrected using multiple hooks instead of a surgical retractor. He showed no permanent deficits, such as an ipsilateral cerebral infarction, although transient postoperative hoarseness was observed. This case report documented a significantly twisted ICA with luminal stenosis, successfully treated via CEA, by correcting the carotid position using multiple hooks with gentle manipulation.

## Introduction

Carotid endarterectomy (CEA) is an established surgical treatment for symptomatic patients with moderate to severe carotid stenosis or asymptomatic patients with severe carotid stenosis ^(1), (2)^. Preoperative evaluation is conducted to check for anatomical variations, specifically at the carotid bifurcation level. A “twisted internal carotid artery (ICA)” is an anatomical variation, wherein the ICA runs medially to the external carotid artery (ECA) at the level of the common carotid artery (CCA) bifurcation ^(3)^. The incidence of a “twisted ICA” varied between 3.6% and 15.1% ^(3), (4), (5), (6), (7), (8), (9)^. The angle of twisting among patients, treated via CEA, ranged from 6° to 95° ^(3), (9)^. This study reports a case of symptomatic severe carotid artery stenosis with significant ICA twisting (102°), successfully managed with CEA.

## Case Report

**Figure 1.**
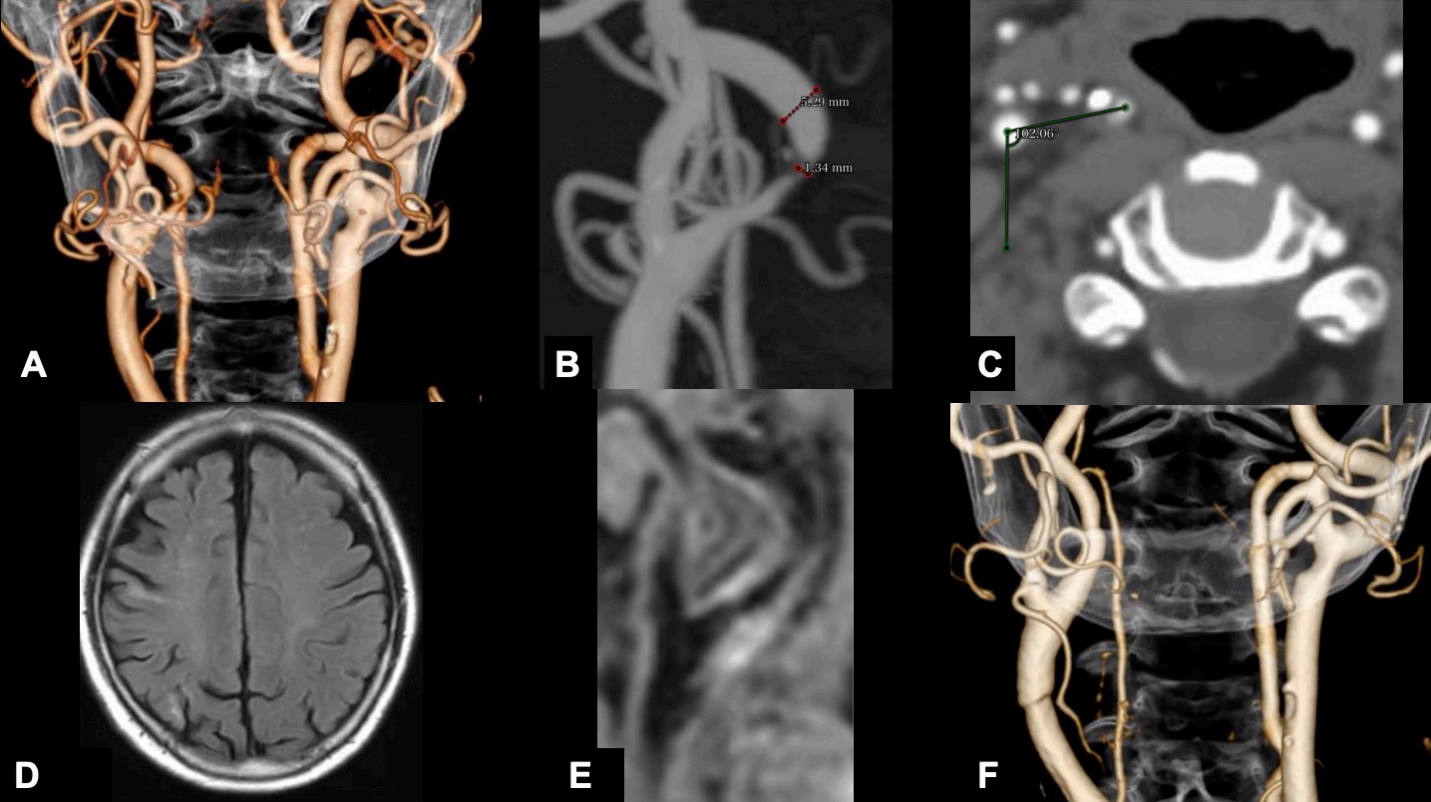
(A) Anteroposterior three-dimensional computed tomography angiography (3D-CTA) showed the internal carotid artery (ICA) medially to the external carotid artery (ECA). (B) CTA showed 74% ICA stenosis according to North American Symptomatic Carotid Endarterectomy Trial criteria. (C) The angle of twisting was measured in the axial view on CTA by computing the degree of medial deviation from the line connecting the centers of the ICA and ECA to the line parallel to the sagittal plane at the center of the ECA. The preoperative twist angle was 102°. (D) Fluid-attenuated inversion recovery imaging showed a chronic cerebral infarction in the right parietal lobe. (E) Black-blood T1-weighted image showed a high-intensity signal, indicating an unstable vulnerable plaque. (F) Postoperative anteroposterior 3D-CTA showed improvement of the ICA stenosis without repositioning.

**Figure 2.**
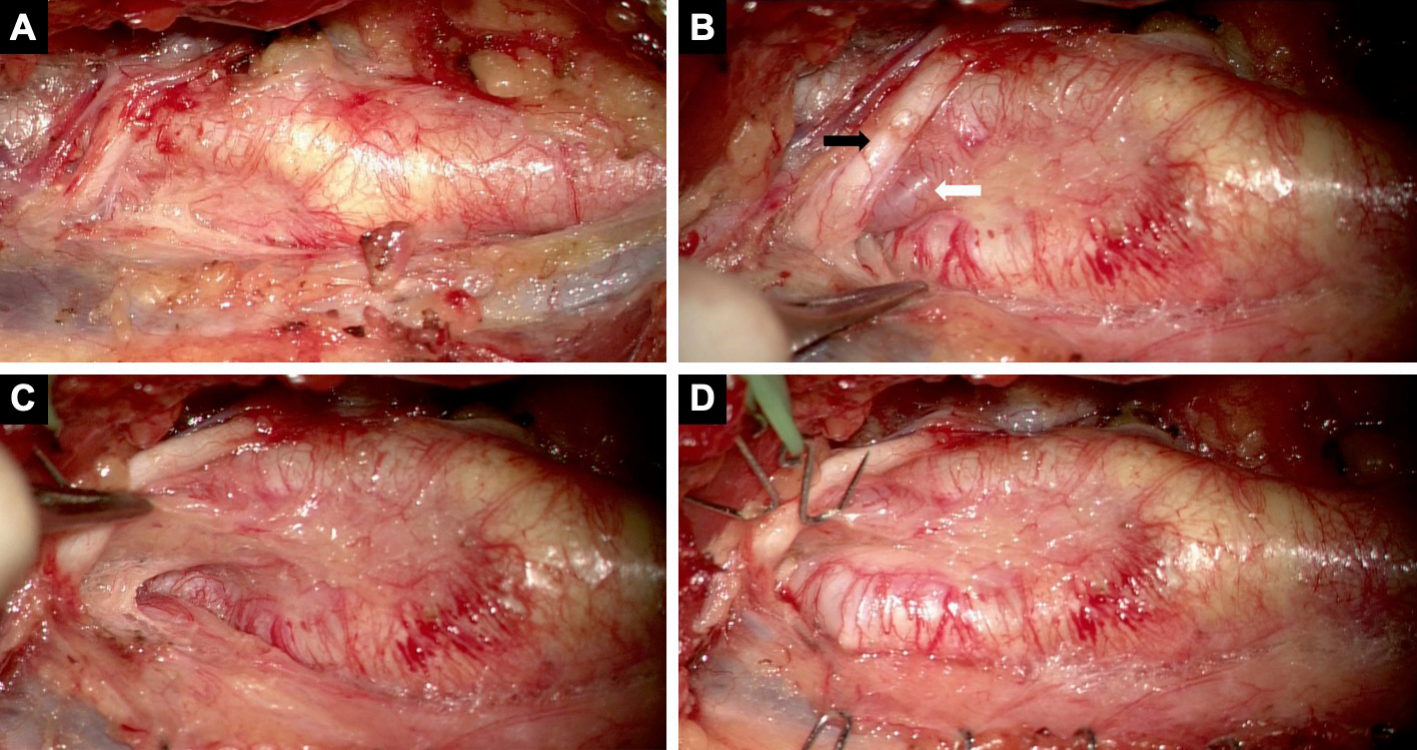
Operative view of the case. (A) The intraoperative photograph at the level of the carotid bifurcation showed the concealed ICA. (B) The ICA was located under the hypoglossal nerve (black arrow) and occipital artery (white arrow). (C) After mobilizing the hypoglossal nerve, the distal ICA ran deeply. (D) After transposing the ICA to its normal location, this surgical field resembled a conventional CEA.

An 81-year-old male with a medical history of myocardial infarction, chronic kidney disease, and hypertension was incidentally diagnosed with asymptomatic carotid artery stenosis. Doppler ultrasonography demonstrated an elevated peak systolic velocity in the right ICA, exceeding 200 cm/s, suggesting severe luminal stenosis. Meanwhile, a three-dimensional computed tomography angiography (3D-CTA) demonstrated 74% stenosis of the ICA, according to the North American Symptomatic Carotid Endarterectomy Trial criteria. Magnetic resonance (MR) imaging revealed an asymptomatic cerebral infarct in the right parietal lobe. No tandem steno-occlusive changes were observed on MR angiography. An unstable carotid plaque was detected on the black-blood MR T1-weighted image (Figure 1A-E). 3D-CTA revealed a significantly twisted ICA, located medial to the ECA, at the level of the CCA bifurcation, proximal to the pharynx. The twist angle, as defined elsewhere ^(9)^, reached 102°. CEA was deemed feasible due to the unstable plaque characteristics, severe stenosis, and anatomical variation of the carotid bifurcation. The significant twisting of the ICA was addressed by repositioning it with wide longitudinal carotid exposure and gentle maneuvering. The operation was performed under general anesthesia with near-infrared spectroscopy monitoring and intraoperative heparin administration, without discontinuing his daily low-dose oral aspirin. He was positioned supine with his neck extended and his face turned 30° contralaterally. Figure 2 shows that the ICA was pulled, and the twisted carotid bifurcation was corrected using multiple hooks attached to a rubber band. The subsequent conventional procedures, such as arterial cross-clamping, arteriotomy, internal shunt tube insertion, meticulous dissection of the carotid plaque, and primary closure with a running suture under an operative microscope, were performed without difficulty. There was no postoperative cerebral infarction. Postoperative CTA showed a twisted ICA, similar to the preoperative findings (Figure 1F). He developed transient mild hoarseness on the third postoperative day. He was discharged on the seventh postoperative day, and the follow-up after one month showed improvement of the hoarseness.

## Discussion

This case report documented a significantly twisted ICA (102°) ^(3), (9)^, successfully treated with CEA, by correcting the twisted carotid bifurcation.

Anatomical variants, such as the “lateral ECA,” “complete transposition of the carotid bifurcation,” “twisted carotid bifurcation,” and “twisted ICA ^(3), (4), (5), (6), (7), (8), (9)^,” were risk factors for postoperative complications of CEA ^(4)^. However, multiple reports have supported the safety of performing CEA in patients with a twisted ICA ^(3), (5), (6), (7), (8), (9)^. The definition of a twisted ICA remains vague; therefore, Katano et al. advocated a standardized method of measuring the twist angle using the axial view of 3D-CTA. Based on this system, the twist angle was equal to the medially deviated degree from the line connecting the centers of the ICA and ECA to the line parallel to the sagittal plane at the center of the ECA ^(9)^. The previously reported twist angles ranged from 6° to 95°, as summarized in the Table 1 ^(3), (9)^. In the present case, the twist angle was 102°. Thus far, this was the highest degree of a twisted ICA. CEA application in patients with a twisted ICA has been documented ^(3), (7), (9)^. In this study, the significantly twisted ICA was corrected, and CEA was performed without difficulty. When correcting the twisted ICA, gentle manipulation is performed to prevent a distal embolism from an unstable carotid plaque or laryngeal nerve injury ^(3), (5), (6), (8)^. However, it was difficult to assess the degree of care during manipulation. A recent study demonstrated a novel and objective assessment of the gentleness of surgical manipulation, using a surgical video of CEA ^(10)^. In the present case, he developed transient postoperative hoarseness, most likely due to the operative procedure or tracheal intubation. This innovative assessment allowed reflecting on the steps of CEA to clarify the cause of postoperative hoarseness. In conclusion, this study described a case of a significantly twisted ICA, treated with CEA. Patients with a significantly twisted ICA are eligible for CEA after the ICA is transposed to its normal location via gentle manipulation.

**Table 1.** Summary of the Previously Reported Twist Angle for the Twisted Internal Carotid Artery.

Case	n	Age (years)	M/F	R/L	Stenosis	Twist evaluation	Twisted angle	Minimum	Maximum
Katano	7	70.4 ± 7.3	3/4	6/1	78.7 ± 10.5	CTA	80.0 ± 17.6	45°	95°
Honda	8	77.0 ± 2.6	8/0	8/0	66.9 ± 19.9	CTA	30.1 ± 17.9	6°	54°
Present case	1	81	1/0	1/0	74	CTA	102°

N: number of cases, M/F: Male/Female, R/L: Right/Left, CTA: computed tomography angiography

## Article Information

### Conflicts of Interest

None

### Acknowledgement

The authors thank Editage [http://www.editage.com] for editing and reviewing this manuscript in English.

### Author Contributions

 Conception of the case report: Oki S, Ito M, and Gekka M; acquisition of data: Oki S, Gekka M, and Yamauchi T; drafting the work: Oki S and Ito M; and critical revision for important intellectual content: Ito M and Fujimura M

### Approval by Institutional Review Board (IRB)

The institutional ethics review board approved this case report (2022-41). The patient provided informed consent for the publication of the case details.

### Disclaimer

Miki Fujimura is one of the Editors of JMA Journal and on the journal’s Editorial Staff. He was not involved in the editorial evaluation or decision to accept this article for publication at all.
